# The Effect of Different Adjuvants on Immune Parameters and Protection following Vaccination of Sheep with a Larval-Specific Antigen of the Gastrointestinal Nematode, *Haemonchus contortus*

**DOI:** 10.1371/journal.pone.0078357

**Published:** 2013-10-21

**Authors:** David Piedrafita, Sarah Preston, Joanna Kemp, Michael de Veer, Jayne Sherrard, Troy Kraska, Martin Elhay, Els Meeusen

**Affiliations:** 1 School of Biomedical Sciences, Monash University, Clayton, Victoria, Australia; 2 The ARC Centre of Excellence in Structural and Functional Microbial Genomics, Monash University, Clayton, Victoria, Australia; 3 Veterinary Medicine Research and Development, Pfizer Animal Health, Parkville, Victoria, Australia; Instituto Butantan, Brazil

## Abstract

It has recently been recognised that vaccine adjuvants play a critical role in directing the nature of a vaccine induced effector response. In the present study, several adjuvants were evaluated for their ability to protect sheep after field vaccination with the larval-specific *Haemonchus contortus* antigen, HcsL3. Using a suboptimal antigen dose, aluminium adjuvant was shown to reduce the cumulative faecal egg counts (cFEC) and worm burden by 23% and 25% respectively, in agreement with a previous study. The addition of Quil A to the aluminium-adjuvanted vaccine brought cFEC back to control levels. Vaccination with the adjuvant DEAE-dextran almost doubled the protection compared to the aluminium-adjuvanted vaccine resulting in 40% and 41% reduction in cFEC and worm counts compared to controls. Examination of skin responses following i.d. injection of exsheathed L3, revealed that cFEC was negatively correlated with wheal size and tissue eosinophils for the DEAE-dextran and aluminium-adjuvanted groups respectively. These studies have for the first time shown the potential of DEAE-dextran adjuvant for helminth vaccines, and discovered significant cellular correlates of vaccine-induced protection.

## Introduction

The generation of natural immunity against gastrointestinal nematode (GIN) parasites, and helminth parasites in general, displays some unique characteristics compared to viral and bacterial infections, in particular in the recruitment and activation of ‘allergic’ or type-2 effector cells (mast cells and eosinophils) [1], [2], [3]. Attempts to generate subunit vaccines against GIN parasites have in the past relied heavily on successes achieved with microbial vaccines, including the use of potent vaccine adjuvants that generate high antibody responses, the major correlates of protection in most existing vaccines [4]. While helminth vaccines based on the ‘hidden antigen’ approach i.e. not boosted by natural immunity, may also rely on high antibody titres [5], it is likely that vaccination strategies aimed at mimicking and accelerating natural immunity will require the induction of both cellular and humoral immunity including the induction of a type-2 effector response.

Vaccine adjuvants have received increased attention in recent years with the realisation that they are the main drivers of both the magnitude and type of adaptive response generated after vaccination [6], [7]. For helminth vaccines aimed at replicating natural immunity, a type-2 immune response may be essential to achieve protection. Indeed, in a previous small pen trial, we observed that immunization with a purified, larval-specific surface antigen of *H. contortus*, was only protective when administered with the type-2 adjuvants, aluminium hydroxide and cholera toxin, while addition of pertussis toxin increased antibody titres but abrogated protection [8].

In the present study, we performed a more extensive trial using 3 different adjuvants currently in use in veterinary vaccines, and determined both the levels of protection and the immune response generated. In order to be compatible with farm management practices, vaccinations were performed on pasture using only two subcutaneous immunizations. A preliminary dose-response trial established significant high levels (61–69%) of protection with the largest antigen dose and a significant but lower 27% protection in the second highest dose group using aluminium hydroxide as the adjuvant [9]. To assess the capacity of different adjuvants to improve protection and conserve antigen, the second highest antigen dose was used in the present study. The results of the study established that one rarely used adjuvant improved protection over aluminium adjuvant, while another more widely used type-1 adjuvant abrogated protection.

Assessment of vaccine efficacy based on infection trials is costly and labour intensive and the availability of more amenable immune correlates of protection is desirable for the development and validation of most vaccines [10]. Antibody levels are at present the only known correlates of vaccine-induced immunity, however no correlation with antibodies and protection has been observed with the current larval-specific vaccine [8], [9]. In the present study, we also examined different immune parameters after vaccination and discovered significant immune correlates of protection in the two vaccinated groups of sheep that showed reduced egg counts and worm burdens.

## Materials and Methods

### Preparation and formulation of the vaccine

A surface extract was prepared from exsheathed L3 as described previously [9]. Briefly, L3 were exsheathed with CO2, resuspended in PBS and placed in a boiling water bath for 15 min. Larvae were pelleted by centrifugation and the supernatant, containing the surface extract, was depleted of small MW molecules and concentrated in one step using 50 kD cut-off Centricon Centrifugal Filter Units (Millipore). This resulted in one dominant, typically broad 75–90 kD band on PAGE, weakly staining with coomassie but reacting strongly with the HcsL3-specific mAb Hc22. As the antigen stained very poorly with coomassie (and not at all with silver stain) accurate weight estimation was not possible and vaccine doses were therefore expressed as L3-equivalents. Each antigen vaccinated sheep received an antigen dose extracted from 20,000 L3 equivalents, a suboptimal dose previously resulting in a small but still significant 27% reduction in cumulative faecal egg count (cFEC) using the adjuvant aluminium hydroxide [9].

Immunization setup is summarised in Table 1. Three groups of 10 sheep each were vaccinated with antigen added to aluminium adjuvant with (Group 2) or without (Group 4) addition of Quil A (Brenntag Nordic, Frederikssund, Denmark), or with antigen added to DEAE-dextran adjuvant (DD) (Sigma-Aldrich) (group 3). A separate group of sheep (Group 1, control) received the aluminium adjuvant without antigen. As preliminary *in vitro* experiments (not shown) established strongest binding of the antigen to aluminium phosphate (AlPO_4_), this preparation was chosen instead of the aluminium hydroxide used in previous experiments [8], [9], and prepared in house by mixing 4.73 mL of 35% w/v AlCl_3_ with 17.04 mL of 25% w/v Na_3_PO_4_.12H_2_0 and 0.47 mL of 30% w/v NaOH, adjusted to final volume with water. The aluminium phosphate was adjusted to a concentration of 1 mg/ml aluminium with sterile PBS (pH 7.2) in the final vaccination dose and mixed thoroughly with (Groups 2 &4) or without (control Group1) the antigen on an automated rotator for 1 h at room temperature (25°C). Quil A (2 mg/ml) was adjusted to a concentration of 1 mg/ml with sterile PBS (pH 7.2) in the final vaccination dose and added to the antigen/AlPO_4_ preparation before mixing (Group 2). DEAE-dextran (DD) (20%w/v; pH 6.8) was adjusted to a concentration of 100 mg/ml with sterile PBS (pH 7.2) in the final vaccination dose and mixed thoroughly with the antigen as described above. Each vaccine dose was contained in 1 ml solution. Enough vaccine was prepared for 2 immunization doses, and the second dose was stored at 4°C until used.

**Table 1:** Sheep numbers, immunization protocols and levels of protection against *H.contortus* infection.

Group #	Sheep numbers (n)	Adjuvant	Antigen (*Hc*sL3) L3 equivalents	cFEC; % protection relative to control	Mean worm burdens; % protection relative to control
1 (Control)	10	AlPO_4_	0	-	-
2	10	AlPO_4_ + Quil A	20 K	3	ND
3	10	DEAE-dextran(DD)	20 K	40^*^	41^**^
4	10	AlPO_4_	20 K	23	25

ND: not done; *p<0.05, **p<0.001.

### Experimental animals, immunization and challenge protocol

Merino-cross wethers were raised and maintained on pasture at a Woodend farm (Northern Victoria). At 8 months of age (week -14), forty sheep were selected and treated with a long-acting anthelmintic, Cydectin®, to remove any existing parasites. Only low egg counts were detected throughout the grazing period (<100 eggs per gram) and no egg counts were detected after treatment. At week -8, sheep were randomised and allocated to 4 experimental groups (n = 10) based on stratified body weight ranking, bled for pre-vaccination serum and given their first immunization dose by subcutaneous injection in the neck region. Four weeks later (week – 4), they were given their second immunization and bled one week later (week -3) for the post-vaccination serum. Two weeks after the second immunization (week -2), they were transported and housed in a large indoor shed at the Monash University experimental Werribee farm. After two weeks acclimatization (week 0), they were infected twice with 7000 L3 on day 0 and day +3 using the *H. contortus* strain, Haecon-5. This strain was isolated in 2006 from the field by Novartis Animal Health, and shown to be more pathogenic than the previously used laboratory strains. Two sheep died on pasture and one indoors due to causes unrelated to the trial.

### Parasitological, serological and haematological measurements

Faecal egg counts (FEC) were assessed between 21 to 56 days post infection (dpi) according to the modified McMaster method and expressed as eggs per gram (EPG) faeces. Egg counts were determined for each collection day, and mean cumulative faecal egg counts (cFEC) were calculated by adding all EPG values for each sheep over the whole collection period. Worm burdens were collected from Groups 1, 3 and 4 and worm recovery was performed as described previously [9]. Briefly, the stomach (abomasum) was removed and cut along the greater curvature. Abomasal contents and washings were collected and made up to a volume of 2 L with tap water containing 1% formalin. The solution was vigorously bubbled with air and 10% transferred to glass trays for manual counting of parasites.

Serum samples were collected before vaccination (week -8), one week after the second immunization (week -3), before challenge (week 0) and one week after challenge (week +1). For determining packed cell volume (PCV), blood was collected before (day 0) and 1, 4 and 7 weeks after challenge in 10 ml EDTA tubes and spun in a Haematocrit centrifuge.

Serum anti-HcsL3 antibodies were determined by enzyme linked immunosorbant assay (ELISA) as described previously [8], [9]. Briefly, ELISA plates (Nunc, Denmark) were coated overnight with L3 surface extract in 100 µl carbonate buffer pH 9.6 and incubated with various dilutions of sheep sera. Specific antibody isotype responses were determined by incubating with mAbs against ovine IgG1, IgG2 [11] and IgA (Serotec, Bicester, UK) followed by HRP-conjugated rabbit anti-mouse reagent (DAKO, Denmark) and developed with 3′, 3′, 5′, 5′- tetramethyl-benzidine dihydrochloride hydrate (TMB; Invitrogen, VIC, Australia). Antigen-specific IgE was determined by ELISA of ammonium sulphate-treated serum samples as described previously [12]. Antibody levels were also compared to a separate control group of 8 sheep that had been kept in indoor pens, worm free for 2–3 months (Penned group).

### Intradermal injections and skin responses

Two days before kill, cutaneous hypersensitivity reactions were performed by two intradermal injections of 100 *H. contortus* exsheathed L3 in 100 µl saline in the right inside back leg of the animal. Two injections of saline were administered as a control at two adjacent sites. The skin temperature and wheal size were measured at 2 hours post injection and at 48 hrs, just prior to euthanasia. Skin temperature at the injection site was recorded with a digital infra-red temperature gauge (Kelly supply company, Australia) and wheal size was measured with digital calipers. After gross removal of the injected skin area, a hollow punch was used to cut skin samples of approximately 1 cm^2^ in size which was sufficient to remove the majority of the inflamed tissue surrounding the injection site, and this was divided in 2 equal parts for histology and future RNA extraction. Histology samples were placed in 10% neutral buffered formalin and then embedded in paraffin, sectioned and stained with haematoxylin and eosin by the Monash University Histology Laboratory. Eosinophils in the entire biopsy sections were counted and expressed as the mean number of cells per 1 mm^2^ tissue (+/- SEM).

### Ethics Statement

Handling of animals and experimental procedures were approved by the Monash University Animal Ethics Committee (Ethics # SOBSA/P/2009/44).

### Statistical Analysis

Data were analysed using GraphPad Prism5. Each vaccinated group was compared against the adjuvant control group using Student's t-test with significance set at p<0.05. Values were log transformed before analysis if variations were significantly different. One way ANOVA was used to compare values between all groups. The Spearman's rank correlation coefficient was calculated to determine significant dependence between two variables.

## Results

### Protection levels after challenge infection of sheep vaccinated with HcsL3 administered with different adjuvants.

**Figure 1:**
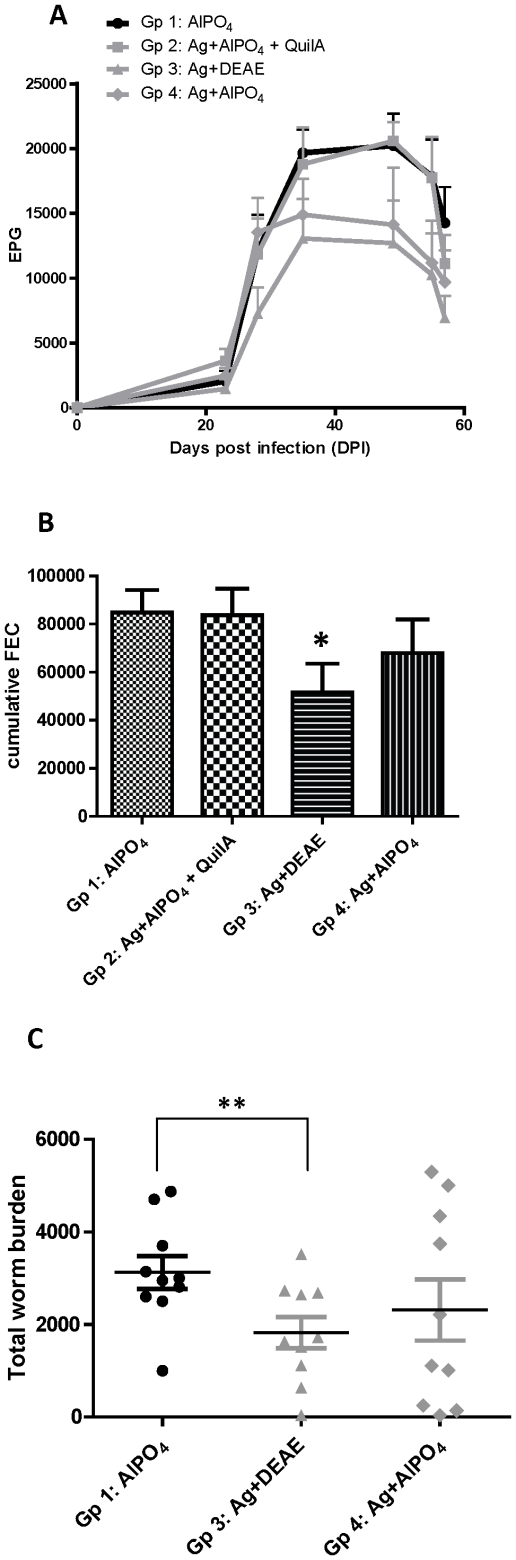
Faecal egg counts and worm burden after challenge of vaccinated sheep. A: Mean (+SEM) daily eggs/gram faeces (EPG); B: Mean (+SEM) cumulative faecal egg count (FEC); C: individual and mean (+/−SEM) total worm counts. For details of experimental groups, refer to Table 1. Significantly different from the control group at p<0.05 (*) or p<0.01 (**).

Eggs per gram faeces (EPG) and cumulative faecal egg counts (cFEC) were reduced in group 4, immunized with antigen and aluminium adjuvant, compared to the control group 1 (Fig.1A&B). The degree of protection (23%, Table 1) was similar to that observed previously with this antigen dose (27%, [9]), although levels were not significant in this case. Addition of Quil A to the same vaccine (group 2) increased egg counts to control levels (Fig.1A&B). Group 3 which received DD as the adjuvant showed the best protection, which was almost double that of group 4 (40%) and reached significance compared to the control group 1 (Table 1). To ensure that the increased protection in group 3 was not due to the DD adjuvant only, in a separate infection trial (not shown) a group of 10 sheep injected with DD alone were compared with a non-injected control group; no difference was observed in the cFEC of these groups, with cFEC of 23893 (SEM ±7628) and 23420 (SEM ±6693), respectively.

Post-mortem worm counts were performed on the control group 1 and the two vaccinated groups 3 and 4 (Fig. 1C). Mean worm counts in group 3 were significantly reduced compared to the control group (p = 0.008). Individual worm counts in group 4 showed wide segregation, indicative of responder and non-responder sheep resulting in no significant difference of the mean compared to group 1. Protection levels for worm counts were similar to the cFEC at 41% and 25% for groups 3 and group 4, respectively (Table 1).

**Figure 2:**
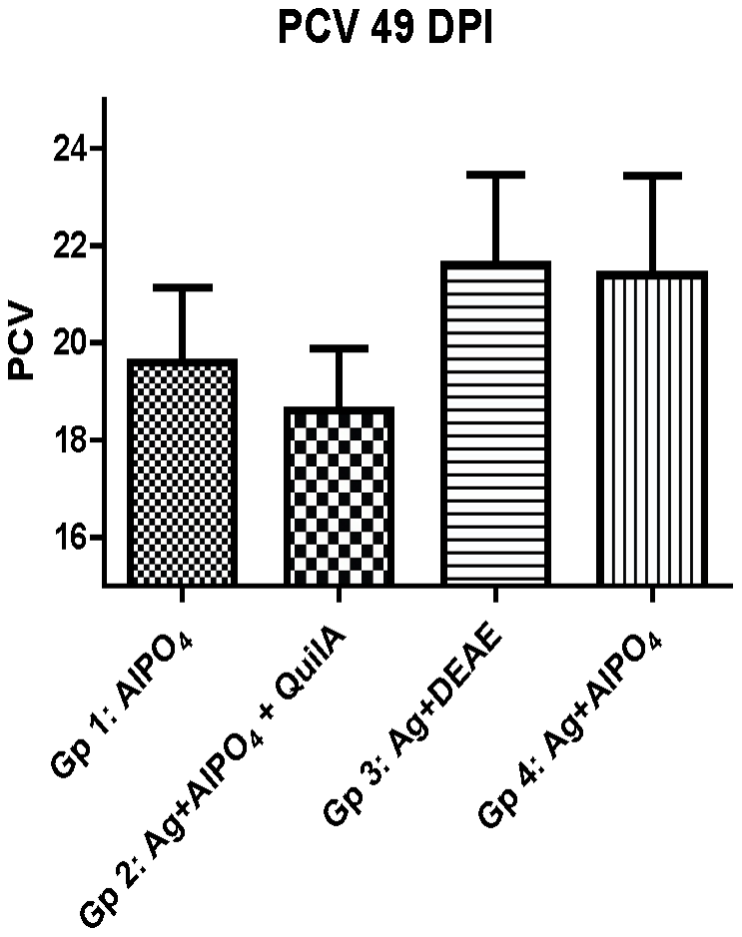
Packed cell volume (PCV) of peripheral blood after challenge of vaccinated sheep. Mean (+SEM) PCV of groups at 49 days post infection (DPI). The mean PCV of all sheep before infection (day 0) was 34.2 (SD 2.3). For details of experimental groups, refer to Table 1.

All groups showed a decrease in PCV values after infection but the mean PCV values at the end of the experiment were similar in groups 3 and 4 and, although not significant, higher than those in groups 1 and 2 (Fig. 2).

### Antibody responses during vaccination and challenge

**Figure 3:**
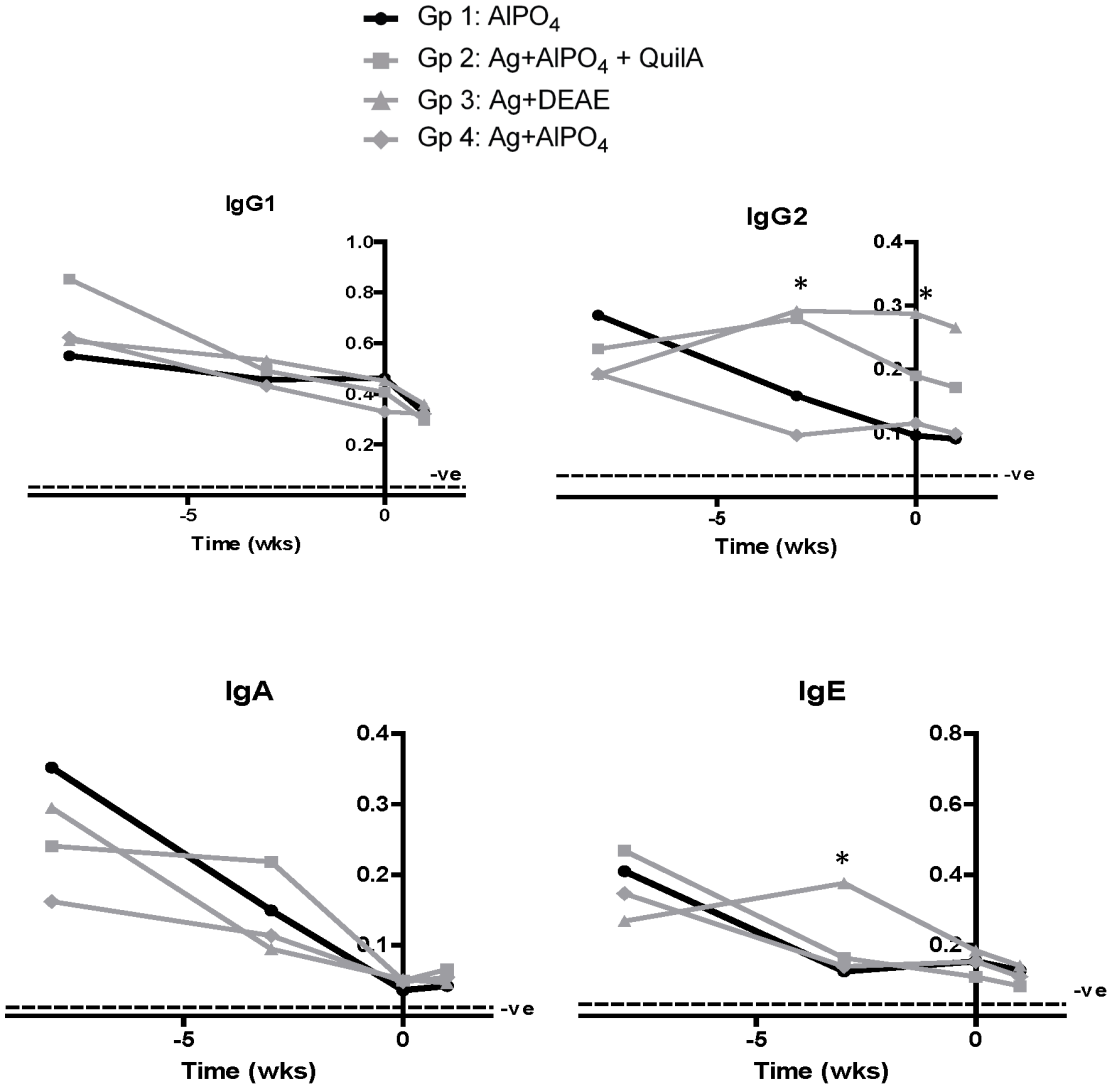
Serum antibody responses after challenge of vaccinated sheep. Mean antibody isotype responses of sheep before vaccination (wk -8), 1 week after vaccination (wk -3), at day of challenge (wk 0) and 1 week after challenge (wk +1). Serum diluted 1/5000 (IgG1), 1/500 (IgG2), 1/20 (IgA) or 1/32 ammonium sulphate cut (IgE). For details of experimental groups, refer to Table 1. Dashed line represents the mean of a separate group of 8 sheep kept in indoor pens for 2–3 months. *Significant difference between group 3 and control group 1.

As observed previously [9], antibody levels overall were elevated in pasture reared sheep compared to penned sheep even before the start of vaccination, and levels decreased in the control and most vaccinated groups when transferred to indoor pens (Fig. 3). Only the DD group showed significantly increased antibody levels 1 week after the second vaccination compared to the unvaccinated control group for the IgG2 and IgE isotypes (Fig. 3). No increase in antibody levels was detectable after the challenge infection.

### Skin responses after intradermal injection of saline or L3 larvae

**Figure 4:**
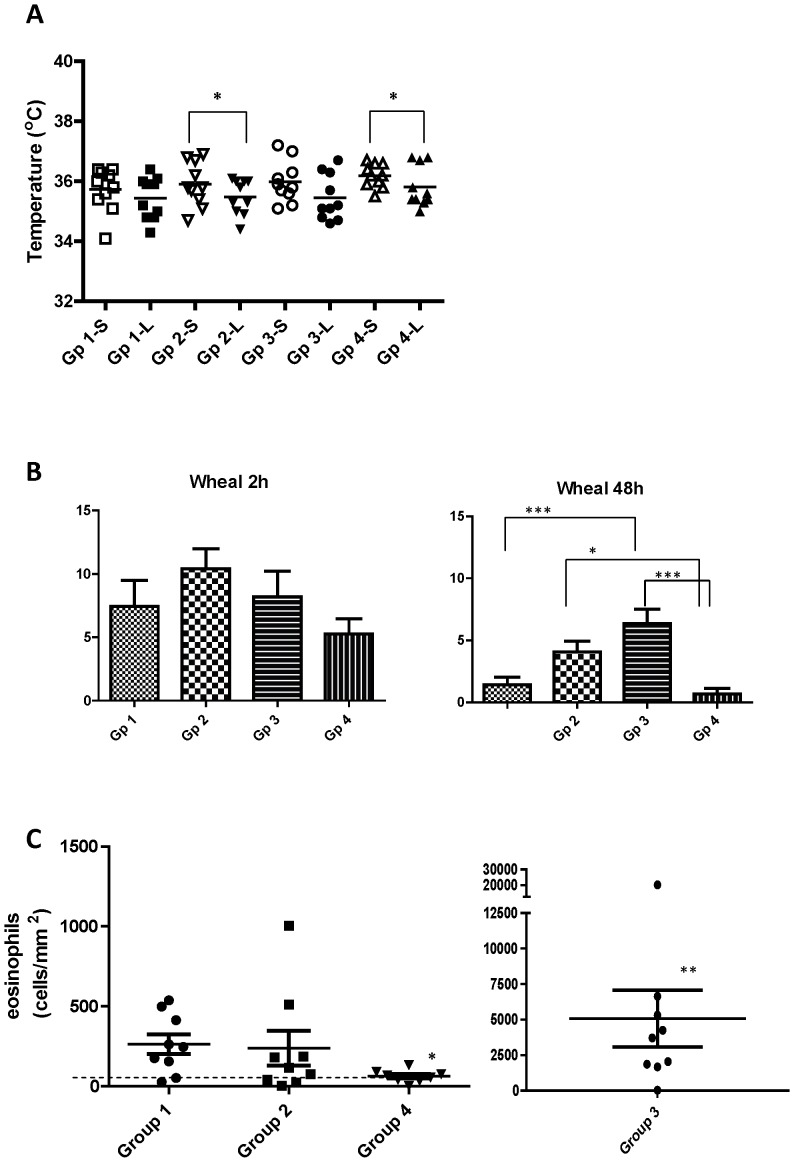
Skin responses after intradermal injection of saline or exsheathed L3 *H. contortus* larvae (xL3). A: temperature of the injected skin sites measured 2 h after intradermal injection of saline (S) or xL3 (L). B: wheal responses measured 2 h or 48 h after intradermal injection of xL3. C: Number of eosinophils/mm^2^ tissue section of xL3 injected sites. Dashed line delineates the upper 95% confidence interval from the mean of the combined saline injected sites. For details of experimental groups, refer to Table 1. Asterisk denotes significance levels at p<0.05 (*), p<0.01 (**) or p<0.001 (***).

Skin temperatures at 2 h were consistently lower in the L3 compared to the saline injected sites (Fig.4A), and this was significant for the two aluminium adjuvant-vaccinated groups (Groups 2 and 4).

Wheal sizes were elevated in all groups at 2 h with no significant difference between groups (Fig.4B). Wheal sizes had decreased significantly by 48 hrs in groups 1, 2 and 4 but remained at a higher level in group 2 compared to group 4. Group 3 showed only a small decrease in wheal size from 2 to 48 h, and its 48 h wheal size was significantly higher than groups 1 and 4.

**Figure 5:**
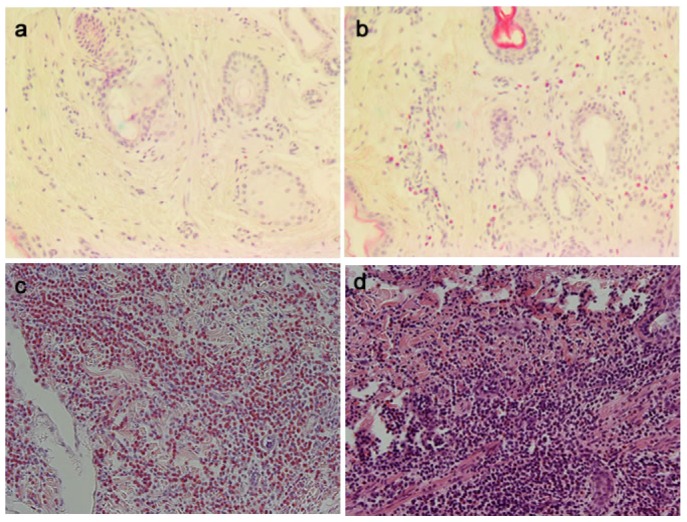
Eosinophil infiltration after intradermal injection of saline or exsheathed L3 *H. contortus* larvae (xL3). Representative H&E stained sections of skin tissue injected with saline (a) or xL3 (b) of group 1,2&4 sheep. Group 3 sheep (Ag+DEAE-dextran) showed much higher eosinophil infiltration (c) and occasional granulomas (d).

There was no difference in eosinophil counts between the control and vaccinated groups in biopsies taken 48 h after saline injection, with a combined mean of 21±9.3 eos/mm^2^. After injection of L3, skin eosinophil counts increased in all groups compared to the saline injections (Fig.4C). This was most pronounced in the DD group 3, where numerous eosinophils were present, often aggregated in large granulomas (Fig. 5) which were not included in the counts. One sheep in the aluminium adjuvant vaccinated group 4 showed a much higher eosinophil count (2969±801) then the other sheep in this group, and with this outlier excluded, mean eosinophil counts in group 4 were significantly below the control group 1 (Fig. 4C).

### Correlations between protection, antibody levels and skin responses

The Spearman's rank correlation coefficients were calculated for all parasitological and immunological measurements, and correlations that showed significance detailed in Table 2. cFEC were significantly correlated with worm counts across all groups examined, and negatively correlated with PCV values. There was no significant correlation between cFEC and any of the antibody isotype levels, except for a slight negative correlation with IgE when all groups were combined. There were however significant but distinct correlations between skin responses and cFEC in the two vaccinated groups with lowest egg counts, groups 3 and 4. Group 3 showed a highly significant negative correlation of cFEC with the 2 h wheal response, while cFEC in group 4 were highly positively correlated with changes in skin temperature at 2 h and negatively correlated with eosinophil counts 48 h after i.d. injection of L3 larvae. The vaccinated group 2 (AlPO_4_+Quil A) also showed a lower but significant negative correlation between cFEC and L3 eosinophil counts.

**Table 2:** Correlations between parasitological and immunological parameters in control and vaccinated sheep.

	Group1: AlPO_4_; n = 10	Group 2: Ag+AlPO_4_+QuilA; n = 10	Group 3: Ag+DEAE-dextran n = 10	Group 4: Ag+AlPO n = 10	All Groups n = 30/40
**Correlations with cumulative faecal egg counts (cFEC)**					
cFEC vs. worm burden	0.78* (0.01)	ND	**0.66 (0.04)**	**0.78 (0.01)**	**0.77 (<0.001)**
cFEC vs. PCV	**−0.68 (0.03)**	−0.60 (0.07)	**−0.76 (0.01)**	**−0.83 (0.003)**	**−0.74 (<0.001)**
cFEC vs. IgE (wk 0)	−0.048 (0.90)	−0.57 (0.09)	−0.09 (0.80)	−0.09 (0.80)	**−0.40 (0.01)**
cFEC vs. wheal (2 h)	−0.003 (0.99)	0.22 (0.54)	−**0.82 (0.004)**	0.032 (0.93)	−0.13 (0.41)
cFEC vs. eos/mm2 (L3)	−0.28 (0.46)	**−0.78 (0.02)**	0.37 (0.34)	**−0.82 (0.01)**	−0.30 (0.13)
cFEC vs. temp change(saline/L3)	0.34 (0.34)	0.05 (0.90)	0.16 (0.66)	**0.77 (0.01)**	0.29 (0.07)
**Correlations of antibody isotypes 1 week after 2^nd^ vaccination (wk -3) and skin responses after i.d. injection of L3**					
wheal 2 h vs. IgE	0.08 (0.83)	0.38 (0.28)	0.28 (0.43)	0.32 (0.37)	0.22 (0.17)
wheal 2 h vs. IgG2	−0.20 (0.58)	0.59 (0.07)	**0.64 (0.047)**	0.47 (0.17)	**0.37 (0.02)**
wheal 48 h vs. IgE	0.04 (0.90)	0.33 (0.35)	**0.83 (0.003)**	0.63 (0.067)	**0.70 (<0.001)**
wheal 48 h vs. IgG1	−0.43 (0.22)	0.48 (0.16)	**0.65 (0.04)**	−0.10 (0.80)	**0.39 (0.01)**

* Spearman rank correlation coefficient (P value); bold numbers indicate significance at P<0.05. ND: not done.

IgG2, but not IgE, correlated significantly with wheal size at 2 h in group 3 and when all groups were combined. At 48 h, this correlation was lost and replaced by significant correlations with IgE and IgG1, again only in group 3 or when all groups were combined.

## Discussion

Recent advances in innate immunity have revealed the critical role adjuvants and vaccine delivery systems play in directing the strength and nature of a vaccine response [6], [7]. In particular, innate receptors, including Toll-like receptors (TLRs) have received wide attention as potential new targets for incorporation into vaccine formulations [13]. However, in most cases of currently used adjuvant systems, the exact innate stimulation pathways are unknown and may not involve TLR activation [6]. This is the case for the most commonly used aluminium adjuvants [14], as well as the two other adjuvants used in the present study, DEAE-dextran (DD) and Quil A. As adjuvants may act differently in different species [15], it is critical to test each experimental vaccine with different adjuvants in the target species.

Vaccination in the current study was performed with a larval-specific antigen and the protective effect is therefore likely to manifest against the early L3 stage during the first 1–2 days after infection, before it moults to an L4. The local response to challenge infections in the gastrointestinal tract is however difficult to study without sacrificing the animals. An attempt was therefore made to replicate the vaccine-induced response against L3 larvae by intradermal injections of exsheathed L3 and subsequent examination of the injection sites. This was done after establishment of the challenge infection so as not to compromise the vaccine efficacy study. Inflammation is generally associated with an increase in temperature and it was therefore surprising that temperature measurements at the L3 injected sites were consistently lower than in the saline injected sites, and this difference was significant in the two groups vaccinated with aluminium adjuvant. This may be related to the findings that type-2 immune responses induced by helminth extracts or aluminium adjuvants dampen type-1 immune responses and pro-inflammatory cytokines [16], [17].

Aluminium adjuvants have been used in most vaccines since the early 1900 and have a proven immune boosting and safety record [6], [14]. The exact action of aluminium adjuvant has not been completely elucidated but seems to be associated with its unique interaction with dendritic cells both *in vitro* [18] and *in vivo* [19]. Aluminium adjuvants are known to bias the immune response towards a type-2 phenotype, including the recruitment of eosinophils, which is generally not considered favourable for bacterial or viral vaccines [14], [17]. However, for protection against helminth parasites, a type-2 response may be advantageous, as eosinophil killing of helminth larvae is a prominent feature of natural immunity in these infections [20]. In particular, eosinophil-mediated killing of L3 *H. contortus* larvae has been shown both *in vitro* [21] and *in vivo* [22]. Surprisingly, eosinophil numbers were not increased but slightly reduced in skin biopsies after injection with L3 in the aluminium vaccinated sheep compared to the adjuvant control group. Eosinophil numbers did however correlate strongly with protection in the aluminium vaccine group, suggesting that they are involved in larval killing as observed during natural infection [20], [22]. *In vitro* studies, have shown that activation of eosinophils is required for effective killing [21] and this may occur in the aluminium vaccinated but not in the control sheep. In addition, as eosinophils in immune sheep are strongly attracted to the tissue larvae [20], [22], it is possible that cell counts of surrounding tissues may not adequately reflect the true level of eosinophil recruitment.

Quil A is a quintessential type-1 adjuvant currently incorporated into several veterinary vaccines [23]. In agreement with the type-2 hypothesis above and its known downregulation by type-1 cytokines, any protection observed in the aluminium adjuvant group was abrogated by the addition of Quil A to the vaccine. Previous studies using pertussis toxin or Freund's complete adjuvant in the HcsL3 vaccine, while increasing the antibody responses, have also resulted in a lack of protection or even exacerbation of infection [8], [24]. Together, these studies emphasise the importance of choosing the right adjuvant for a particular disease or a desired immune effector response.

DEAE-dextran (DD) is a high molecular weight positively charged polymer that has received renewed interest as a vaccine adjuvant due to its antibody enhancing properties in anti-fertility vaccines [25]. The mode of action and the type of immune response elicited with this adjuvant have however not been studied in detail. In the present study, DD was the only adjuvant that elicited a detectable increase in antigen-specific antibodies, and these were significant for the IgG2 and IgE isotypes. In addition, skin biopsied after i.d. L3 injection revealed a massive increase in eosinophils, orders of magnitude above the control and aluminium injected sheep, indicating a predominant type-2 immune response induced by this adjuvant. Eosinophil numbers in skin biopsies taken 48 h after i.d. L3 injection, did not however correlate with protective immunity in this group, but were difficult to accurately enumerate due to their large numbers and the presence of numerous eosinophil-rich granulomas. Protection in the DD group of sheep was significantly correlated with the 2 h wheal responses suggesting that the protection observed in this group was manifest at this early time period and eosinophil numbers at this time may show correlation with protection. It is also possible that other cell types such as neutrophils and monocytes may have been recruited and contributed to protection and this requires further detailed studies at the earlier time points.

The present study has confirmed the critical dependence of effective vaccines against infections on the nature of the immune response induced by a particular adjuvant system. For helminth vaccines that are based on mimicking natural immunity against invading larvae, this may involve the use of adjuvants that induce a type-2 immune response, most likely involving eosinophil-mediated killing. In contrast to most bacterial and viral vaccines where protection is based on antibodies only, the present vaccine has shown no correlation with antibody levels, but significant correlations with cellular responses, in particular eosinophil recruitment and/or activation. It is however likely that specific antibodies will still be required for effective antibody-dependent cell mediated killing of invading larvae [20]. In addition to the protection observed previously with aluminium adjuvant, the results of this study have also provided a more effective adjuvant system for helminth vaccines and revealed significant correlates of vaccine-induced protection.
